# Restenosis and risk of stroke after stenting or endarterectomy for symptomatic carotid stenosis in the International Carotid Stenting Study (ICSS): secondary analysis of a randomised trial

**DOI:** 10.1016/S1474-4422(18)30195-9

**Published:** 2018-07

**Authors:** Leo H Bonati, John Gregson, Joanna Dobson, Dominick J H McCabe, Paul J Nederkoorn, H Bart van der Worp, Gert J de Borst, Toby Richards, Trevor Cleveland, Mandy D Müller, Thomas Wolff, Stefan T Engelter, Philippe A Lyrer, Martin M Brown, International Carotid Stenting Study investigators, Ale Algra, S J Bakke, Neil Baldwin, Jonathan Beard, Christopher Bladin, J Martin Bland, J Boiten, Mark Bosiers, A W Bradbury, David Canovas, Brian Chambers, Angel Chamorro, Jonathan Chataway, Andrew Clifton, Rory Collins, Lucy Coward, Anna Czlonkowska, Stephen Davis, L DeJaegher, David Doig, Paul Dorman, Jörg Ederle, Roland F Featherstone, Jose M Ferro, Peter Gaines, G Gilling-Smith, M Goertler, A Gottsäter, Werne Hacke, Alison Halliday, George Hamilton, J M H Hendriks, Michael Hill, L Jaap Kapelle, Markku Kaste, Fiona Kennedy, P Konrad, LJS Kool, Peter J Koudstaal, I Malik, Hugh Markus, Peter Martin, Jean-Louis Mas, Charles McCollum, T McGahan, A J McGuire, Philippe Michel, Andrew Molyneux, Jane Moroney, A Mosch, J Moss, Ross Naylor, A Peeters, D Roy, David Schultz, D M Seriki, R A Shinton, Paul Sidhu, J Stewart, G Subramanian, R Sztajzel, P G Than, Daffyd Thomas, E Turner, J S P van den Berg, G Vanhooren, Graham Venables, Nils Wahlgren, S Walker, Charles Warlow, Bojana Zvan

**Affiliations:** a Stroke Research Centre, Department of Brain Repair and Rehabilitation, Institute of Neurology, University College London, London, UK; b Department of Neurology and Stroke Center, University Hospital Basel, University of Basel, Basel, Switzerland; c Department of Vascular Surgery, University Hospital Basel, University of Basel, Basel, Switzerland; d Department of Medical Statistics, London School of Hygiene & Tropical Medicine, London, UK; e Department of Neurology, Stroke Service, and Vascular Neurology Research Foundation, The Adelaide and Meath Hospital, Dublin, incorporating the National Children's Hospital, Dublin, Ireland; f Academic Unit of Neurology, School of Medicine, Trinity College Dublin, Dublin, Ireland; g Department of Clinical Neurosciences, Institute of Neurology, London, UK; h Department of Neurology, Academic Medical Center Amsterdam, Amsterdam, Netherlands; i Department of Neurology and Neurosurgery, Brain Center Rudolf Magnus, University Medical Center Utrecht, Utrecht, Netherlands; j Department of Vascular Surgery, University Medical Center Utrecht, Utrecht, Netherlands; k Division of Surgery and Interventional Science, University College London, London, UK; l Sheffield Vascular Institute, Sheffield Teaching Hospitals NHS Foundation Trust, Sheffield, UK; m Neurorehabilitation Unit, University Center for Medicine of Aging and Rehabilitation, Felix Platter Hospital, Basel, Switzerland

## Abstract

**Background:**

The risk of stroke associated with carotid artery restenosis after stenting or endarterectomy is unclear. We aimed to compare the long-term risk of restenosis after these treatments and to investigate if restenosis causes stroke in a secondary analysis of the International Carotid Stenting Study (ICSS).

**Methods:**

ICSS is a parallel-group randomised trial at 50 tertiary care centres in Europe, Australia, New Zealand, and Canada. Patients aged 40 years or older with symptomatic carotid stenosis measuring 50% or more were randomly assigned either stenting or endarterectomy in a 1:1 ratio. Randomisation was computer-generated and done centrally, with allocation by telephone or fax, stratified by centre, and with minimisation for sex, age, side of stenosis, and occlusion of the contralateral carotid artery. Patients were followed up both clinically and with carotid duplex ultrasound at baseline, 30 days after treatment, 6 months after randomisation, then annually for up to 10 years. We included patients whose assigned treatment was completed and who had at least one ultrasound examination after treatment. Restenosis was defined as any narrowing of the treated artery measuring 50% or more (at least moderate) or 70% or more (severe), or occlusion of the artery. The degree of restenosis based on ultrasound velocities and clinical outcome events were adjudicated centrally; assessors were masked to treatment assignment. Restenosis was analysed using interval-censored models and its association with later ipsilateral stroke using Cox regression. This trial is registered with the ISRCTN registry, number ISRCTN25337470. This report presents a secondary analysis, and follow-up is complete.

**Findings:**

Between May, 2001, and October, 2008, 1713 patients were enrolled and randomly allocated treatment (855 were assigned stenting and 858 endarterectomy), of whom 1530 individuals were followed up with ultrasound (737 assigned stenting and 793 endarterectomy) for a median of 4·0 years (IQR 2·3–5·0). At least moderate restenosis (≥50%) occurred in 274 patients after stenting (cumulative 5-year risk 40·7%) and in 217 after endarterectomy (29·6%; unadjusted hazard ratio [HR] 1·43, 95% CI 1·21–1·72; p<0·0001). Patients with at least moderate restenosis (≥50%) had a higher risk of ipsilateral stroke than did individuals without restenosis in the overall patient population (HR 3·18, 95% CI 1·52–6·67; p=0·002) and in the endarterectomy group alone (5·75, 1·80–18·33; p=0·003), but no significant increase in stroke risk after restenosis was recorded in the stenting group (2·03, 0·77–5·37; p=0·154; p=0·10 for interaction with treatment). No difference was noted in the risk of severe restenosis (≥70%) or subsequent stroke between the two treatment groups.

**Interpretation:**

At least moderate (≥50%) restenosis occurred more frequently after stenting than after endarterectomy and increased the risk for ipsilateral stroke in the overall population. Whether the restenosis-mediated risk of stroke differs between stenting and endarterectomy requires further research.

**Funding:**

Medical Research Council, the Stroke Association, Sanofi-Synthélabo, and the European Union.

## Introduction

Endarterectomy and stenting aim to lower the long-term risk of stroke in patients with atherosclerotic disease of the carotid artery. Findings of randomised trials of these procedures for treatment of symptomatic carotid stenosis show that, by comparison with endarterectomy, stenting has a higher risk of stroke, particularly in older patients, but has lower risks of myocardial infarction, cranial nerve palsy, and access-site haematoma.^1^, ^2^, ^3^, ^4^ Beyond the initial periprocedural period, stenting and endarterectomy were equally effective at preventing recurrent stroke, but findings on long-term patency of the treated carotid artery after each procedure have been conflicting.^5^, ^6^, ^7^, ^8^, ^9^, ^10^ Importantly, the question of whether residual or recurrent stenosis after treatment increases the risk of recurrent stroke has not been answered definitively.

**Research in context**

**Evidence before this study**

We searched the Cochrane Stroke Group Trials Register, the Cochrane Central Register of Controlled Trials, MEDLINE, Embase, and Science Citation Index up to April, 2016, for randomised controlled trials published in the English language comparing carotid artery stenting versus carotid endarterectomy in patients with symptomatic or asymptomatic carotid stenosis. Only trials using primary carotid stenting in their endovascular treatment group—ie, with routine placement of a stent—were included. We compared the odds of at least moderate (≥50% narrowing of the lumen) carotid restenosis or occlusion, and of severe (≥70%) restenosis or occlusion. The trials used different ultrasound criteria to measure stenosis severity. Eight trials have reported severe (≥70%) restenosis, three of which additionally reported at least moderate (≥50%) restenosis. Only one trial showed a significantly increased incidence of severe (≥70%) restenosis after stenting than after endarterectomy, whereas in the other seven trials no difference was noted. At least moderate (≥50%) restenosis occurred significantly more often after stenting than after endarterectomy in two trials (one of which was very small), whereas in another very small trial no difference was noted. In a systematic review, the presence of severe (≥70%) carotid restenosis or occlusion increased the risk of stroke after endarterectomy but not after stenting. However, whether stroke risk is already increased at lower degrees of restenosis (ie, ≥50%) remained unclear.

**Added value of this study**

The International Carotid Stenting Study (ICSS) is the largest randomised trial reporting long-term restenosis of various severity and subsequent risks of stroke after stenting versus endarterectomy for treatment of symptomatic carotid stenosis. The results of the current analysis show that moderate or higher (≥50%) restenosis is significantly more frequent after stenting compared with endarterectomy, but severe (≥70%) restenosis rates did not differ. These findings accord with previous data. ICSS is the first trial to show that the presence of at least moderate (≥50%) restenosis increases the risk for subsequent ipsilateral stroke and for stroke in any territory. This increased stroke risk was only significant in the endarterectomy group.

**Implications of all the available evidence**

Carotid artery stenting is associated with a higher long-term risk for moderate or higher restenosis (leading to 50% or more luminal narrowing) than is endarterectomy. Carotid restenosis increases the risk for stroke, but this risk gain might be more pronounced after endarterectomy than after stenting. Further evidence is needed to assess the usefulness of regular follow-up of patients after carotid revascularisation with duplex ultrasound and to ascertain whether repeat revascularisation is beneficial in those with restenosis.

The International Carotid Stenting Study (ICSS) is the largest randomised trial to date comparing stenting with endarterectomy for symptomatic carotid stenosis. We previously reported results up to 10 years after randomisation, which showed that each procedure was equally effective at preventing fatal or disabling stroke—the primary outcome measure of the trial.^9^ Moreover, we reported no difference in the long-term risk of severe (≥70%) restenosis after either procedure.

The aims of this prespecified secondary analysis of ICSS were to quantify the long-term risk of at least moderate (≥50%) restenosis up to 10 years after randomisation, to ascertain whether restenosis predisposed to a higher risk of subsequent stroke after either procedure, and to investigate the risk factors predisposing to restenosis. We postulated that moderate or higher (≥50%) restenosis would be more frequent after stenting than endarterectomy and might predispose to a higher risk of recurrent stroke during long-term follow-up.

## Methods

### Participants

ICSS included patients with carotid stenosis associated with ipsilateral transient ischaemic attack or stroke symptoms within the 12 months before inclusion. We recruited patients from 50 tertiary care centres in Europe, Australia, New Zealand, and Canada (appendix). The trial was approved by the Northwest Multicentre Research Ethics Committee in the UK. Individual participating centres also obtained site-specific approval from their local research ethics committees. All patients provided written informed consent to participate in the trial before randomisation. Details on centre requirements, patients' eligibility criteria, randomisation, and treatment have been published previously.^3^, ^9^, ^11^ The trial protocol is available on the trial website.

### Randomisation and masking

In brief, patients aged 40 years or older with symptomatic atherosclerotic carotid stenosis (≥50% diameter reduction), who were judged by local investigators equally suited for both treatments, were randomly assigned to treatment by stenting or endarterectomy in a 1:1 ratio. Randomisation was done by telephone or fax using a computerised service provided by the Oxford Clinical Trials Service Unit, which was not involved in other aspects of the trial. Randomisation was stratified by centre and was minimised for sex, age, side of stenosis, and occlusion of the contralateral carotid artery. Patients and investigators were aware of the treatment assignment.

### Procedures

Carotid imaging was done before randomisation to confirm the diagnosis of stenosis measuring 50% or more. The diagnosis was made by either selective digital subtraction angiography or concordant findings on extracranial carotid duplex ultrasound and non-invasive angiography (magnetic resonance angiography [MRA] or computed tomography angiography [CTA]). The interventionist used their discretion to choose stents and cerebral protection devices, but they had to be CE-marked and approved by the Trial Steering Committee. Surgeons could do either standard or eversion endarterectomy, under local or general anaesthesia, with or without the use of shunts or patches. We judged stenting complete when a stent was placed across the stenosis, and we deemed endarterectomy complete when the plaque was removed and the arteriotomy wound closed. All patients received medical care including antiplatelet therapy or anticoagulation, if indicated, and control of vascular risk factors.

We followed up patients at 30 days after treatment then at 6 months after randomisation and annually thereafter. The duration of follow-up was initially planned for 5 years but was extended to 10 years after randomisation in patients able and willing to continue.

The protocol specified that carotid duplex ultrasound was to be done at every follow-up visit. Peak systolic velocities in the common carotid artery and internal carotid artery, and end diastolic velocity in the internal carotid artery were recorded on duplex ultrasound and reported to the central trial office. One investigator (LHB)—who was masked to treatment allocation and date of follow-up—graded stenosis according to predefined criteria that equate well with stenosis severity measured on catheter angiography, using the North American Symptomatic Carotid Endarterectomy Trial (NASCET) method of estimating stenosis.^12^ To quantify the severity of stenosis, we used a cutoff for peak systolic velocity in the internal carotid artery greater than 1·3 m/s for at least moderate (≥50%) stenosis and greater than 2·1 m/s for severe (≥70%) stenosis. We also considered the end diastolic velocity in the internal carotid artery and the ratio of peak systolic velocities in the internal carotid artery to the common carotid artery (appendix).^13^ No correction was made for the presence of a stent when measuring stenosis, based on the results of a study of a subset of patients treated with stents in ICSS, which showed similar estimates of stenosis severity from simultaneous CTA and duplex ultrasound examinations.^14^ Ultrasound velocity measurements were not available from a few study centres; in these cases, we used the percentage stenosis reported by the local ultrasonographer and investigator. Additional carotid ultrasound and other imaging studies (eg, MRA or CTA) could be done if needed outside the regular follow-up intervals—eg, if patients had recurrent cerebrovascular events. In these cases, the same investigator (LHB) quantified the degree of stenosis by the NASCET method and these results were also included in the present analysis.

### Outcomes

The primary outcome of ICSS was fatal or disabling stroke, and this outcome has been reported previously.^9^ The protocol of ICSS specified stenosis greater than 70% or occlusion during follow-up as a secondary outcome measure. For this study, we defined moderate or higher (≥50%) restenosis as stenosis of the treated carotid artery measuring 50% or greater, including occlusion, seen at any time during follow-up after completion of treatment. We defined severe (≥70%) restenosis as any stenosis of 70% or greater, or occlusion. The definition of restenosis did not distinguish between recurrent stenosis and residual stenosis present immediately after treatment.

An independent endpoint committee masked to treatment allocation adjudicated major clinical outcome events. In the present analysis, we investigated whether restenosis caused ipsilateral stroke of any severity—ie, occurring in the territory supplied by the treated carotid artery—and stroke in any vascular territory. We defined stroke clinically as a rapidly developing clinical syndrome of focal retinal or cerebral dysfunction lasting more than 24 h or leading to early death, with no other apparent non-vascular cause.

### Statistical analysis

We did the present analysis per protocol. We only included patients in whom the randomly allocated treatment was initiated and completed and in whom at least one post-procedural ultrasound follow-up examination was done and available for analysis. We excluded patients who did not have revascularisation, those who underwent aborted procedures, and those who crossed over to receive the alternative procedure. We censored patients at the time of any further ipsilateral revascularisation procedure during follow-up, at their last ultrasound examination, or death. We assumed non-informative censoring. We compared time to censoring at the last ultrasound examination between treatment groups using the log-rank test to check whether the duration of ultrasound follow-up was similar in both groups. Because the restenosis outcome was interval-censored (ie, restenosis was only known to have occurred at some point between the previous ultrasound scan and the one showing restenosis), we analysed time to restenosis using a generalised non-linear model, which assumes proportional hazards, and the treatment effect parameter estimate can be interpreted as a log hazard ratio (HR).^13^, ^15^ We tested proportionality of hazards via interactions with follow-up periods.

We used a likelihood ratio test to calculate the treatment effect p value. We calculated HRs for restenosis, with endarterectomy as the reference group, both without adjustment and after adjustment for patients' baseline characteristics associated independently with restenosis. We identified such baseline characteristics using forward stepwise selection in a generalised non-linear model that included treatment as a covariate. In a post-hoc analysis, we additionally checked the association between statin use at 30 days after treatment and restenosis during follow-up (data for statin use at baseline were not gathered in ICSS). We calculated the cumulative incidence of restenosis at 1 year and 5 years after treatment using the Kaplan-Meier method, with time to restenosis set to the midpoint between the previous normal scan and the one showing restenosis. We truncated Kaplan-Meier plots of time to restenosis at 7 years, because the number of patients in whom ultrasound follow-up was continued beyond this timepoint was relatively small.

To investigate the association between occurrence of restenosis and subsequent stroke during follow-up, we compared event hazards between patients without restenosis (starting from 30 days after the initial revascularisation procedure) and those with restenosis (beginning at the first scan showing restenosis), using Cox proportional hazards models. We used a time-updated covariate,^16^ whereby a patient with restenosis was included in the no restenosis group up until the scan showing restenosis, thereafter they were in the restenosis group. We counted strokes occurring before the scan showing restenosis in the no restenosis group (appendix). We censored patients at the time of their stroke and disregarded further ultrasound scans. We calculated HRs of outcome events, with no restenosis as the reference group, without adjustment and again with adjustment for baseline characteristics associated independently with restenosis. We did this analysis for both moderate or higher (≥50%) restenosis and severe (≥70%) restenosis.

We did all statistical analyses in Stata 14.1 and used the intcens command for models for interval-censored data. All reported p values are two-sided, with a value less than 0·05 judged significant. We made no adjustment for multiple comparisons.

This study is registered with the ISRCTN registry, number ISRCTN25337470.

### Role of the funding source

The funder had no role in study design, data collection, data analysis, data interpretation, or writing of the report. LHB, JG, JD, and MMB had full access to all data in the study. The corresponding author had final responsibility for the decision to submit for publication.

## Results

**Figure 1:**
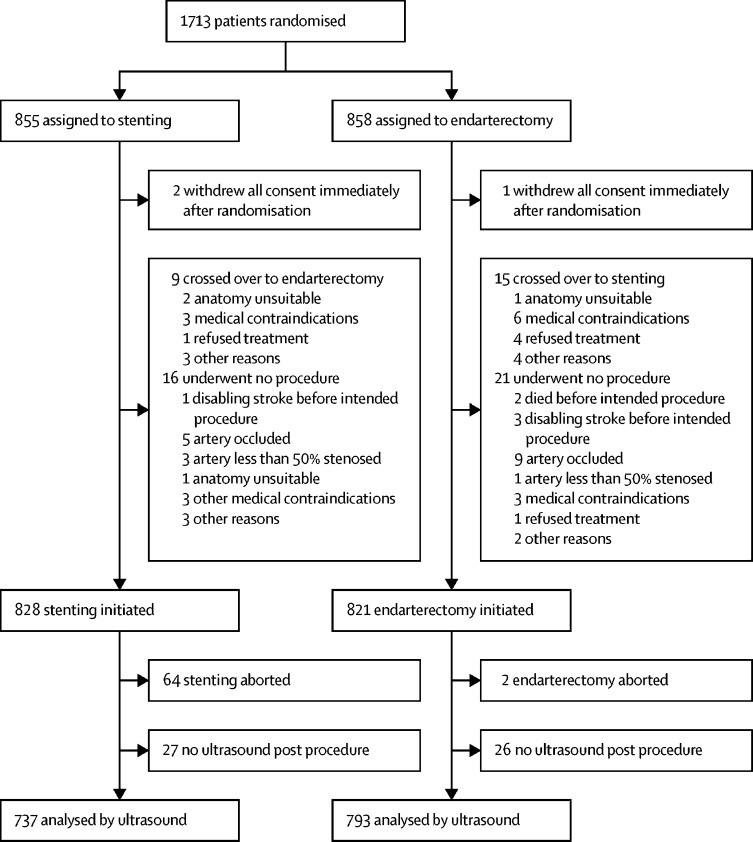
Trial profile

Between May, 2001, and October, 2008, 1713 patients were recruited to ICSS, of whom 855 were randomly assigned to stenting and 858 to endarterectomy (figure 1). Ultrasound follow-up was done in 1530 (97%) of 1583 patients who completed treatment, 737 in the stenting and 793 in the endarterectomy groups, with a median duration of follow-up of 4·0 years (IQR 2·3–5·0) and a maximum follow-up of 10 years. No difference was noted in the duration of ultrasound follow-up between the two treatment groups. Baseline characteristics of patients included in the restenosis analysis were similar in the two treatment groups and to the full ICSS trial population (table 1).

**Table 1:** Baseline characteristics

		**Stenting group**		**Endarterectomy group**	
		Included in analyses	All patients	Included in analyses	All patients
**Demographics**					
Age (years)		70·0 (63·6–76·5)	70·8 (64·4–76·9)	70·6 (64·1–76·9)	70·8 (64·1–76·9)
Men		513/737 (70%)	601/853 (70%)	561/793 (71%)	606/857 (71%)
Women		224/737 (30%)	252/853 (30%)	232/793 (29%)	251/857 (29%)
**Vascular risk factors and history**					
Treated hypertension		503/730 (69%)	587/843 (70%)	552/787 (70%)	596/851 (70%)
Systolic blood pressure (mm Hg)		147·4 (24·1)	147·0 (24·0)	146·0 (23·7)	146·0 (23·6)
Diastolic blood pressure (mm Hg)		79·3 (11·8)	79·2 (11·7)	78·2 (12·6)	78·3 (12·7)
Diabetes		162/737 (22%)	184/853 (22%)	165/793 (21%)	188/857 (22%)
	Non-insulin dependent	116/730 (16%)	134/843 (16%)	128/787 (16%)	147/851 (17%)
	Insulin dependent	46/730 (6%)	50/843 (6%)	37/787 (5%)	41/851 (5%)
Treated hyperlipidaemia		459/730 (63%)	522/843 (62%)	523/787 (66·5)	563/851 (66%)
Total cholesterol (mmol/L)		4·9 (1·3)	4·8 (1·3)	4·9 (1·3)	4·9 (1·3)
Current smoker		182/730 (25%)	205/843 (24%)	183/787 (23%)	198/851 (23%)
Ex-smoker		352/730 (48%)	408/843 (48%)	392/787 (50%)	424/851 (50%)
Angina in past 6 months		70/730 (10%)	83/843 (10%)	69/787 (9%)	77/851 (9%)
Previous myocardial infarction		126/730 (17%)	151/843 (18%)	145/787 (18%)	156/851 (18%)
Previous coronary-artery bypass graft		95/730 (13%)	109/843 (13%)	106/787 (13%)	116/851 (14%)
Atrial fibrillation		44/730 (6%)	57/843 (7%)	52/787 (7%)	59/851 (7%)
Other cardioembolic source		15/730 (2%)	19/843 (2%)	16/787 (2%)	16/851 (2%)
Cardiac failure		21/730 (3%)	23/843 (3%)	40/787 (5%)	47/851 (6%)
Peripheral artery disease		123/730 (17%)	139/843 (16%)	122/787 (16%)	136/851 (16%)
Degree of symptomatic carotid stenosis*					
50–69%		75/737 (10%)	92/853 (11%)	71/793 (9%)	76/857 (9%)
70–99%		662/737 (90%)	761/853 (89%)	722/793 (91%)	781/857 (91%)
Degree of contralateral carotid stenosis*					
<50%		495/737 (67%)	565/853 (66%)	522/793 (66%)	561/857 (65%)
50–69%		104/737 (14%)	128/853 (15%)	131/793 (17%)	142/857 (17%)
70–79%		93/737 (13%)	105/853 (12%)	99/793 (12%)	110/857 (13%)
Occluded		43/737 (6%)	49/853 (6%)	35/793 (4%)	37/857 (4%)
Unknown		2/737 (<1%)	6/853 (1%)	6/793 (1%)	7/857 (1%)
**Most recent ipsilateral event before randomisation**†					
Retinal ischaemia (amaurosis fugax or retinal infarct)		157/728 (22%)	174/840 (21%)	153/781 (20%)	165/844 (20%)
Transient ischaemic attack		239/737 (32%)	273/853 (32%)	280/793 (35%)	303/857 (35%)
Ischaemic hemispheric stroke		332/737 (45%)	393/853 (46%)	348/793 (44%)	376/857 (44%)
Unknown		9/737 (1%)	13/853 (2%)	12/793 (2%)	13/857 (2%)
Time from event to procedure (days)		35 (15–82)	35 (15–82)	40 (18–87)	40 (18–87)
Treatment within 14 days of event		181/737 (25%)	206/837 (25%)	144/791 (18%)	151/834 (18%)

Data are either median (IQR), number of patients/total number (%), or mean (SD). Some totals do not add up to 100% because of rounding.

* Degree of stenosis reported by randomising centre according to the measure used in the North American Symptomatic Carotid Endarterectomy Trial or a non-invasive equivalent.

† If two events were reported on the same day, the more severe event was counted.

**Figure 2:**
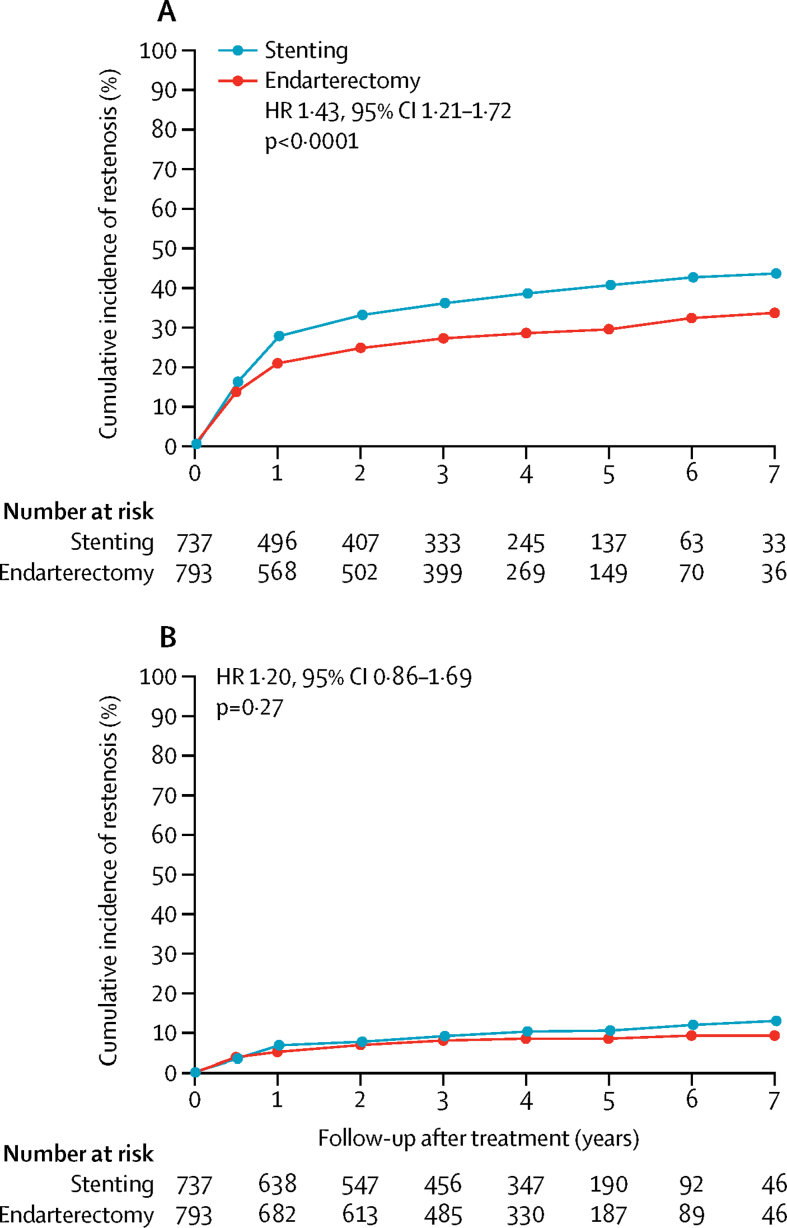
Cumulative incidence of (A) at least moderate (≥50%) carotid artery restenosis or occlusion and (B) severe (≥70%) carotid artery restenosis or occlusion after completed treatment Cumulative incidence was estimated by life-table analysis. Plots stop at 7 years' follow-up because the number of patients at risk beyond that time was fewer than 100, but analyses were based on all follow-up data (maximum 10 years). HR=unadjusted hazard ratio.

At least moderate (≥50%) restenosis occurred more frequently in the stenting group (n=274 patients) than in the endarterectomy group (n=217), with resulting cumulative 5-year risks of 40·7% versus 29·6% (unadjusted HR 1·43, 95% CI 1·21–1·72; p<0·0001; table 2, figure 2A). No difference was noted between the two treatment groups in long-term risk of severe (≥70%) carotid restenosis or occlusion, which occurred in 10·6% of patients in the stenting group and 8·5% of patients in the endarterectomy group in the first 5 years (unadjusted HR 1·20, 95% CI 0·86–1·69; p=0·27; table 2, figure 2B). 29 patients had an occlusion of the treated carotid artery during follow-up, 16 in the stenting group and 13 in the endarterectomy group.

**Table 2:** Carotid artery restenosis or occlusion after stenting compared with endarterectomy

	**Stenting (n=737)**	**Endarterectomy (n=793)**	**Stenting *vs* endarterectomy**			
			Unadjusted HR (95% CI)	p value	Adjusted HR (95%CI)*	p value
**At least moderate (≥50%) restenosis or occlusion**						
Total number of patients with outcome	274	217	1·43 (1·21–1·72)	<0·0001	1·39 (1·14–1·69)	0·001
Cumulative 1-year incidence (95% CI)	27·8% (24·7–31·3)	21·0% (18·3–24·1)	..	..	..	..
Cumulative 5-year incidence (95% CI)	40·7% (36·9–44·8)	29·6% (26·3–33·2)	..	..	..	..
**Severe (≥70%) restenosis or occlusion**						
Total number of patients with outcome	72	62	1·20 (0·86–1·69)	0·27	1·17 (0·83–1·67)	0·36
Cumulative 1-year incidence (95% CI)	6·9% (5·2–9·0)	5·2% (3·8–7·0)	..	..	..	..
Cumulative 5-year incidence (95% CI)	10·6% (8·4–13·3)	8·5% (6·7–10·9)	..	..	..	..

HR=hazard ratio.

* Adjusted for baseline characteristics predictive of restenosis (appendix).

In the forward stepwise selection analysis of variables associated independently with restenosis, associations were very similar in both stenting and endarterectomy groups. Therefore, any association between baseline characteristics and risk of restenosis was analysed in both treatment groups combined. Older age, female sex, current or past smoking, non-insulin dependent diabetes, a history of angina, a greater extent of stenosis in the contralateral carotid artery at randomisation, raised systolic and diastolic blood pressures at randomisation, and higher total serum cholesterol at randomisation increased the risk of restenosis independently of each other and of treatment assignment (appendix). Statin use at 30 days after treatment was not associated with any change in the risk of restenosis. Greater contralateral carotid stenosis and non-insulin dependent diabetes also predicted severe restenosis. HRs for restenosis between stenting and endarterectomy were very similar after adjustment for these independent predictors (table 2).

**Figure 3:**
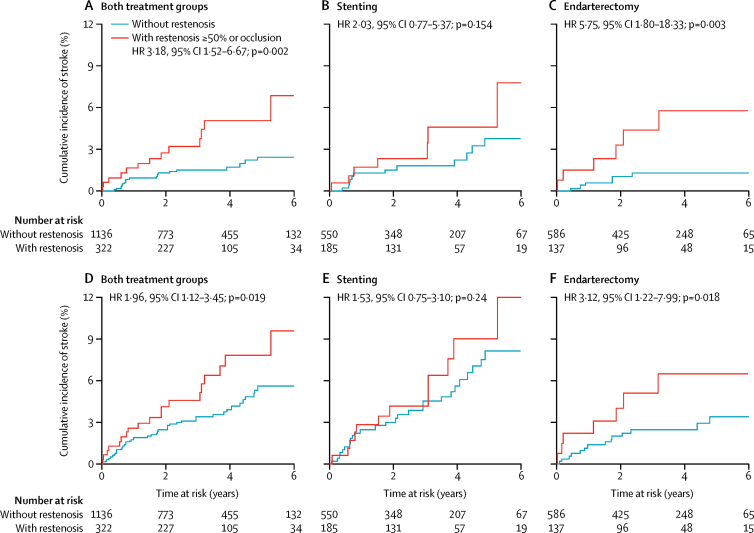
Kaplan-Meier curves of time to (A–C) ipsilateral stroke and (D–F) stroke in any territory with and without at least moderate (≥50%) carotid artery restenosis or occlusion HR=unadjusted hazard ratio.

In both treatment groups combined, patients with at least moderate (≥50%) restenosis were at increased risk of subsequent ipsilateral stroke, with a cumulative risk at 6 years of 6·9% versus 2·5% for patients without restenosis (unadjusted HR 3·18, 1·52–6·67; p=0·002; figure 3A, table 3). 6-year cumulative risk of stroke in any territory was also increased to 9·6% in patients with moderate or higher (≥50%) restenosis versus 5·6% in those without restenosis (1·96, 1·12–3·45; p=0·019; figure 3D, table 3). Analysis by treatment group showed that the restenosis-mediated increase in risk for these events was significant in the endarterectomy group, with a 6-year cumulative risk of ipsilateral stroke of 5·8% in patients with moderate or higher (≥50%) restenosis versus 1·3% in those without restenosis (unadjusted HR 5·75, 1·80–18·33; p=0·003; figure 3C, table 3) and a 6-year risk of any stroke of 6·5% in patients with at least moderate (≥50%) restenosis and 3·4% in those without restenosis (3·12, 1·22–7·99; p=0·018; figure 3F, table 3). No significant increase in risk was noted in the stenting group, with 6-year cumulative risk of ipsilateral stroke of 7·8% in patients with at least moderate (≥50%) restenosis versus 3·8% in those without restenosis (unadjusted HR 2·03, 0·77–5·37; p=0·154; figure 3B, table 3) and risk of any stroke of 12·0% in patients with moderate or higher (≥50%) restenosis versus 8·1% in those without restenosis (1·53, 0·75–3·10, p=0·24; figure 3E, table 3). The formal test of a statistical interaction between type of treatment (stenting or endarterectomy) and the increase in ipsilateral stroke risk caused by restenosis resulted in p=0·10. Associations remained significant after adjustment for patients' baseline characteristics predicting restenosis. The presence of severe (≥70%) restenosis or occlusion was not associated with an increase in subsequent stroke, but the power of this analysis was limited by the few patients with severe (≥70%) stenosis (table 4).

**Table 3:** Association of at least moderate (≥50%) carotid artery restenosis with subsequent outcome events

	**Both groups**		**Stenting**				**Endarterectomy**			
	HR (95% CI)	p value	Events with restenosis (n/N)	Events without restenosis (n/N)	HR (95% CI)	p value	Events with restenosis (n/N)	Events without restenosis (n/N)	HR (95% CI)	p value
**Ipsilateral stroke**										
Unadjusted	3·18 (1·52–6·67)	0·002	7/185	12/550	2·03 (0·77–5·37)	0·154	6/137	6/586	5·75 (1·80–18·33)	0·003
Adjusted*	2·98 (1·39–6·40)	0·005	7/185	12/550	2·06 (0·75–5·63)	0·161	6/137	6/586	5·83 (1·76–19·33)	0·004
**Any stroke**										
Unadjusted	1·96 (1·12–3·45)	0·019	12/185	27/550	1·53 (0·75–3·10)	0·24	7/137	15/586	3·12 (1·22–7·99)	0·018
Adjusted*	1·81 (1·00–3·26)	0·048	12/185	27/550	1·46 (0·70–3·04)	0·312	7/137	15/586	3·48 (1·32–9·15)	0·012

The first 30 days after a procedure, and patients with restenosis in the first 30 days, are excluded from this analysis. Patients were censored at the time of stroke or at any ipsilateral procedure during follow-up. HR=hazard ratio.

* Adjusted for predictors of restenosis (appendix). Only participants with complete information on these baseline risk factors were included in the adjusted comparisons.

**Table 4:** Association of severe (≥70%) carotid artery restenosis with subsequent outcome events

	**Both groups**		**Stenting**				**Endarterectomy**			
	HR (95% CI)	p value	Events with restenosis (n/N)	Events without restenosis (n/N)	HR (95% CI)	p value	Events with restenosis (n/N)	Events without restenosis (n/N)	HR (95% CI)	p value
**Any stroke**										
Unadjusted	1·79 (0·64–4·99)	0·263	3/38	37/569	2·06 (0·63–6·82)	0·234	1/34	23/611	1·34 (0·18–10·10)	0·774
Adjusted*	1·64 (0·58–4·62)	0·346	3/38	37/569	1·96 (0·58–6·59)	0·278	1/34	23/611	1·35 (0·18–10·31)	0·772
**Ipsilateral stroke**										
Unadjusted	1·66 (0·39–7·03)	0·489	1/38	18/569	1·37 (0·18–10·49)	0·761	1/34	13/611	2·20 (0·28–16·99)	0·450
Adjusted*	1·46 (0·34–6·22)	0·613	1/38	18/569	1·25 (0·16–9·80)	0·830	1/34	13/611	1·83 (0·23–14·56)	0·568

The first 30 days after a procedure, and patients with restenosis in the first 30 days, are excluded from this analysis. Patients were censored at the time of stroke or at any ipsilateral procedure during follow-up. HR=hazard ratio.

* Adjusted for predictors of restenosis (appendix). Only participants with complete information on these baseline risk factors were included in the adjusted comparisons.

## Discussion

In this secondary analysis of ICSS, the long-term risk of moderate or higher (≥50%) restenosis, or occlusion of the carotid artery, was significantly higher after stenting than after endarterectomy. As previously reported,^9^ the risk of severe (≥70%) carotid restenosis, or occlusion, did not differ between treatment groups. Among patients in both groups combined, those with at least moderate (≥50%) restenosis had a significantly increased risk of subsequent ipsilateral stroke compared with those without restenosis. Analysed separately, the increase in stroke risk in patients with restenosis compared with those without restenosis was significantly higher after initial treatment with endarterectomy, but not after initial treatment with stenting.

Findings of randomised trials have shown that among patients with symptomatic carotid stenosis, stenting is associated with a greater risk of non-disabling procedural stroke than is endarterectomy, but the long-term efficacy at preventing recurrent stroke after the procedural period is equivalent for each procedure.^5^, ^8^, ^9^, ^10^ Despite these reassuring data for stroke prevention, concerns have remained about the risk of restenosis after treatment and whether restenosis increases the long-term risk of recurrent stroke. In the Carotid and Vertebral Artery Transluminal Angioplasty Study (CAVATAS),^13^ endovascular treatment was compared with endarterectomy in patients with predominantly symptomatic carotid stenosis, using the same criteria for grading restenosis as in ICSS. The cumulative 5-year risk of moderate or higher restenosis (≥50%) was 59% after endovascular treatment and 32% after endarterectomy, and risks of severe restenosis (≥70%) or occlusion were 31% and 11%, respectively (p<0·0001). However, most patients in the endovascular arm of this early trial were treated by balloon angioplasty alone, without insertion of stents. Since then, several trials comparing primary stenting versus endarterectomy for symptomatic carotid stenosis with ultrasound follow-up have published mid-term and long-term outcomes. In the Stent-Supported Percutaneous Angioplasty of the Carotid Artery versus Endarterectomy (SPACE) trial,^5^ rates of severe restenosis (≥70%) 2 years after stenting were significantly higher than after endarterectomy (10·7% *vs* 4·6%; p=0·0009), using ultrasound velocity criteria established at the individual local centres. In the Endarterectomy Versus Angioplasty in Patients With Symptomatic Severe Carotid Stenosis (EVA-3S) trial,^6^ the proportion of patients with moderate or higher (≥50%) restenosis at 3 years—defined by planimetric measurements on ultrasound—was higher after stenting than after endarterectomy (13% *vs* 5%; p=0·02). However, the incidence of velocity-defined severe restenosis (≥70%), using the same cutoff for peak systolic velocity in the internal carotid artery as in our study (≥2·1 m/s), did not differ at either 5 years (stenting 2·3% *vs* endarterectomy 4·2%) or 10 years (5·0% *vs* 8·3%) after treatment.^8^ Likewise, in the Carotid Revascularization Endarterectomy versus Stenting Trial (CREST),^10^ in which 1159 patients with symptomatic and 1032 patients with asymptomatic carotid stenosis were followed up by ultrasound, the combined 10-year incidence of severe carotid restenosis (≥70%, using a cutoff for peak systolic velocity in the internal carotid artery of ≥3·0 m/s) or repeat revascularisation did not differ between the stenting (12·2%) and endarterectomy (9·7%) groups (HR 1·24, 95% CI 0·91–1·70). In a previous analysis by the CREST investigators,^7^ restenosis 3 years after treatment was compared using several cutoffs for peak systolic velocity. Severe restenosis—defined by a peak systolic velocity in the internal carotid artery of 2·1 m/s or higher—was significantly more frequent after stenting than after endarterectomy (14·8% *vs* 10·5%; p=0·02), whereas when a cutoff for peak systolic velocity in the internal carotid artery of 3·0 m/s or greater was used, no difference was noted (6·0% *vs* 6·3%; p=0·58). The frequency of restenosis equivalent to our measure of 50% or greater—ie, a cutoff for peak systolic velocity in the internal carotid artery of greater than 1·3 m/s—was not reported.

Some investigators have recommended using higher cutoffs for peak systolic velocity on duplex ultrasound for a given degree of stenosis in stented carotid arteries compared with native arteries because of possible alterations in arterial wall elasticity after stenting.^17^, ^18^ However, in ICSS, a comparison was done of duplex ultrasound velocities and degree of restenosis determined by CTA in a subset of 103 patients treated with stents.^14^ The optimum cutoff for peak systolic velocity in the internal carotid artery was greater than 1·25 m/s, which is similar to that used in our study for identifying moderate or higher (≥50%) restenosis. Hence, we used the same standardised velocity criteria in both treatment groups in this current analysis. The threshold for peak systolic velocity in the internal carotid artery we used was also similar to one recommended in a consensus paper (>1·25 m/s).^19^

We identified older age, female sex, current or past smoking, non-insulin dependent diabetes, history of angina, greater degree of stenosis in the contralateral carotid artery at randomisation, higher systolic or diastolic blood pressure at randomisation, and higher total serum cholesterol at randomisation as independent predictors of restenosis. In CREST, women, patients with diabetes, and those with dyslipidaemia were also at greater risk of restenosis after either treatment, as were smokers after endarterectomy.^7^ Researchers on the CAVATAS trial also identified smoking as an independent risk factor for restenosis.^13^ Although it is intuitive that vascular risk factors increase the risk for restenosis, the reason why female sex was associated with increased risk of restenosis is uncertain. However, this association might relate to the fact that the diameter of carotid arteries is smaller in women compared with men; thus, a given width of intimal hyperplasia might lead to a greater degree of stenosis in women than in men.^20^

Our finding that at least moderate (≥50%) restenosis on routine ultrasound follow-up conferred an increased risk of stroke over time has important implications. In the CAVATAS trial,^13^ patients who developed severe restenosis (≥70%) within the first year after treatment had a higher risk of subsequent ipsilateral transient ischaemic attack or stroke combined, but no increase in risk for ipsilateral stroke alone. However, the trial had a third fewer patients than in ICSS. CREST also reported a higher risk of ipsilateral, non-procedure-related stroke during up to 4 years of follow-up among patients who developed severe restenosis at any time during the first 2 years after treatment.^7^ However, in some patients, restenosis might only have been diagnosed after the stroke had occurred. Therefore, we chose an alternative approach by comparing the risk from the time of diagnosis of restenosis onwards with the risk in patients who did not develop restenosis.

When we analysed outcomes in each treatment group separately, the increase in stroke risk in patients with restenosis was raised significantly after endarterectomy, but not after stenting. However, the formal test for statistical interaction between the type of treatment and the increase in ipsilateral stroke risk caused by restenosis gave a p value of 0·10, indicating that the difference between endarterectomy and stenting was not significant (using the threshold p<0·05) and, therefore, could have arisen by chance. Nevertheless, the disparity in risk raises the question of whether pathological processes leading to restenosis might differ between operated and stented arteries. Restenosis occurring in the first 2 years after endarterectomy is attributed commonly to neointimal hyperplasia characterised by a proliferation of smooth muscle cells, which was thought to be associated with a low risk of thromboembolic events.^21^, ^22^, ^23^, ^24^ Restenosis occurring later is most likely caused by recurrent atherosclerosis. In ICSS, however, most occurrences of restenosis in both treatment groups arose in the first 2 years and, nonetheless, restenosis increased the risk of stroke in the surgery group. Proliferation of smooth muscle cells has also been noted in a patient with severe restenosis 15 months after balloon angioplasty of the internal carotid artery,^25^ suggesting similarities with post-surgical restenosis, at least in the early phase. It is possible thougAh that the newly formed endothelium on the stent surface confers some protection against thromboembolic events in the case of luminal narrowing.

In a meta-analysis of summary data from several other randomised trials,^26^ the risk of stroke was increased after a diagnosis of severe (≥70%) restenosis after endarterectomy, but not after stenting. This observation of a differential risk corroborates our findings, although the effect of moderate or higher (≥50%) restenosis was not examined. Consistent with the findings of this meta-analysis, the risk of ipsilateral stroke in patients with restenosis after endarterectomy remained relatively low in ICSS, at just over 1% per year. Pooled time-to-event analyses of individual patient-level data from randomised trials could yield further insight into duplex criteria identifying patients with restenosis at higher risk of stroke and the difference in stroke risk associated with restenosis after stenting and endarterectomy.

Our study had some limitations. First, velocity measurements were analysed as recorded by local investigators, and we were not able to review duplex images to check that angle correction was done in all cases. However, our methodology accords with that used in several other studies.^8^, ^13^, ^27^ Second, the true effect of restenosis on risk for recurrent stroke could have been underestimated because restenosis might only have been diagnosed after a stroke occurred, because we used a time-updated covariate model. For the same reason, the effect of severe restenosis (*vs* moderate or higher restenosis) on subsequent stroke could have been underestimated because restenosis might only have been moderate at the time of the last ultrasound scan and could have become severe before the event occurred. Third, our study could have been underpowered to detect a possible difference in severe restenosis between stenting and endarterectomy and an effect of severe restenosis on stroke risk. Fourth, despite the noted increase in stroke risk in patients with restenosis, our data neither allow us to draw firm conclusions on the usefulness of regular ultrasound follow-up after carotid revascularisation nor justify repeat revascularisation in patients with restenosis. A larger sample size is needed to establish treatment-specific ultrasound velocity cutoffs identifying patients at risk for recurrent stroke, which will require a combined analysis of patient-level data from several randomised trials.

In conclusion, moderate or higher (≥50%) restenosis occurred more frequently after stenting than after endarterectomy. Restenosis after revascularisation of the carotid artery increased significantly the risk for subsequent stroke. However, it remains unclear from our results whether this risk is common to both procedures or whether it is significantly more pronounced in patients who undergo endarterectomy.
