# Supplementary material for: Restenosis and risk of stroke after stenting or endarterectomy for symptomatic carotid stenosis in the International Carotid Stenting Study (ICSS): secondary analysis of a randomised trial
Source: Lancet Neurol. 2018 Jul;17(7):587–96. doi: 10.1016/S1474-4422(18)30195-9 (PMC6004555; doi:10.1016/S1474-4422(18)30195-9)
Supplement: Supplementary appendix [file mmc1.pdf]

THE LANCET
Neurology

Supplementary appendix

This appendix formed part of the original submission and has been peer reviewed. We post it as supplied by the authors.

Supplement to: Bonati LH, Gregson J, Dobson J, et al. Restenosis and risk of stroke after stenting or endarterectomy for symptomatic carotid stenosis in the International Carotid Stenting Study (ICSS): secondary analysis of a randomised trial. *Lancet Neurol* 2018; published online May 31. http://dx.doi.org/10.1016/S1474-4422(18)30195-9.

Supplementary Material

International Carotid Stenting Study investigators

Steering Committee:

A Algra, J Bamford (chair), J Beard, M Bland, A W Bradbury, M M Brown (chief investigator), A Clifton, P Gaines, W Hacke, A Halliday, I Malik, J L Mas, A J McGuire, P Sidhu, G Venables.

Credential committee:

A Bradbury, M M Brown, A Clifton, P Gaines.

Data Monitoring Committee:

R Collins, A Molyneux, R Naylor, C Warlow (chair).

Outcome Event Adjudication Committee:

J M Ferro, D Thomas.

Central office staff at UCL Institute of Neurology:

L H Bonati, L Coward, J Dobson (trial statistician), D. Doig, J Ederle, R F Featherstone (trial manager), F. Kennedy, H Tindall, E Turner, D J H McCabe, A Wallis.

Centres participating in ICSS (local investigators):

Australia: Austin Health, Heidelberg (M Brooks, B Chambers [principal investigator], A Chan, P Chu, D Clark, H Dewey, G Donnan, G Fell, M Hoare, M Molan, A Roberts, N Roberts). Box Hill Hospital (Monash University),

Melbourne (B Beiles, C Bladin [principal investigator], C Clifford, G Fell, M Grigg, G New).

Monash Medical Centre, Clayton (R Bell, S Bower, W Chong, M Holt, A Saunder, P G Than [principal investigator]).

Princess Alexandra Hospital, Brisbane (S Gett, D Leggett, T McGahan [principal investigator], J Quinn, M Ray, A Wong, P Woodruff). Repatriation General Hospital, Daw Park, Adelaide

(R Foreman, D Schultz [principal investigator], R Scroop, B Stanley). Royal Melbourne Hospital, Melbourne (B Allard, N Atkinson, W Cambell, S Davis [principal investigator], P Field, P Milne, P Mitchell, B Tress, B Yan). Royal Hobart Hospital, Hobart (A Beasley, D Dunbabin, D Stary, S Walker [principal investigator]).

Belgium: Antwerp University Hospital, Antwerp (P Cras, O d'Archambeau, J M H Hendriks [principal investigator], P Van Schil). A Z St Blasius, Dendermonde (M Bosiers [principal investigator], K Deloose, E van Buggenhout). A Z Sint Jan Brugge-Oostende, Campus Brugge, Brugges (J De Letter, V Devos, J Ghekiere, G Vanhooren [principal investigator]). Cliniques Universitaires St Luc, Bruxelles (P Astarci, F Hammer, V Lacroix, A Peeters [principal investigator], R Verhelst). Imelda Ziekenhuis, Bonheiden (L DeJaegher [principal investigator], A Peeters, J Verbist).

Canada: CHUM Notre-Dame Hospital, Montreal (J-F Blair, J L Caron, N Daneault, M-F Giroux, F Guilbert, S Lanthier, L-H Lebrun, V Oliva, J Raymond, D Roy [principal investigator], G Soulez, A Weill). Foothills Medical Center, Calgary (M Hill [principal investigator], W Hu, M Hudion, W Morrish, G Sutherland, J Wong).

Finland: Helsinki University Central Hospital, Helsinki (A Albäck, S Curtze, H Harno, P Ijäs, M Kaste (principal investigator), K Lappalainen, M Lepäntalo, A Meretoja, S Mustanoja, T Paananen, M Porras, J Putaala, M Railo, T Sairanen, L Soinne, A Vehmas, P Vikatmaa).

Germany: Otto von Guericke University, Magdeburg (M Goertler [principal investigator], Z Halloul, M Skalej).

Ireland: Beaumont Hospital, Dublin (P Brennan, C Kelly, A Leahy, J Moroney [principal investigator], J Thornton).

Netherlands: Academic Medical Center, Amsterdam (M J W Koelemay, P J Nederkoorn [principal investigator], J A A Reekers, Y B W E M Roos). Erasmus Medical Center, Rotterdam (J M Hendriks, P J Koudstaal [principal investigator], P M T Pattynama, A van der Lugt, L C

van Dijk, M R H M van Sambeek, H van Urk, H J M Verhagen). Haga Teaching Hospitals, The Hague (C M A Bruijninckx, S F de Bruijn, R Keunen, B Knippenberg, A Mosch [principal investigator], F Treurniet, L van Dijk, H van Overhagen, J Wever). Isala Klinieken, Zwolle (F C de Beer, J S P van den Berg [principal investigator], B A A M van Hasselt, D J Zeilstra). Medical Center Haaglanden, The Hague (J Boiten [principal investigator], J C A de Mol van Otterloo, A C de Vries, G J Lycklama a Nijeholt, B F W van der Kallen). UMC St Radboud, Nijmegen (J D Blankensteijn, F E De Leeuw, L J Schultze Kool [principal investigator], J A van der Vliet). University Medical Center, Utrecht (G J de Borst, G A P de Kort, L J Kapelle [principal investigator], T H Lo, W P Th M Mali, F Moll, HB van der Worp, H Verhagen).

New Zealand: Auckland City Hospital, Auckland (P A Barber, R Bourchier, A Hill, A Holden, J Stewart [principal investigator]).

Norway: Rikshospitalet University Hospital, Oslo (S J Bakke [principal investigator], K Krohg-Sørensen, M Skjelland, B Tennøe).

Poland: Institute of Psychiatry and Neurology (2nd Department of Neurology & Department of Neuroradiology) and Medical University of Warsaw (2nd Department of General, Vascular and Oncological Surgery), Warsaw (P Bialek, Z Biejat, W Czepiel, A Czlonkowska [principal investigator], A Dowzenko, J Buczek, A Kobayashi, M Lelek, J Polanski).

Slovenia: University Medical Center, Ljubljana (J Kirbis, Z Milosevic, B Zvan [principal investigator]).

Spain: Hospital Clinic, Barcelona (J Blasco, A Chamorro [principal investigator], J Macho, V Obach, V Riambau, L San Roman). Parc Taulí Sabadell Hospital, Barcelona (J Branera, D Canovas [principal investigator], Jordi Estela, A Gimenez Gaibar, J Perendreu).

Sweden: Skåne University Hospital, Malmö (K Björses, A Gottsäter [principal investigator], K Ivancev, T Mätzsch, B Sonesson)). Sodersjukhuset, Stockholm (B Berg, M Delle, J Formgren, P Gillgren, T-B Kaell, P Konrad [principal investigator], N Nyman, R Takolander). The

Karolinska Institute, Stockholm (T Andersson, J Malmstedt, M Soderman, C Wahlgren, N Wahlgren [principal investigator]).

Switzerland: Centre Hospitalier Universitaire Vaudois, Lausanne (S Binaghi, L Hirt, P Michel [principal investigator], P Ruchat). University Hospital Basel, Basel (L H Bonati, S T Engelter, F Fluri, L Guerke, A L Jacob, E Kirsch, P A Lyrer [principal investigator], E-W Radue, P Stierli, M Wasner, S Wetzel). University Hospital of Geneva, Geneva (C Bonvin, A Kalangos, K Lovblad, N Murith, D Ruefenacht, R Sztajzel [principal investigator]).

United Kingdom: Addenbrookes Hospital, Cambridge (N Higgins, P J Kirkpatrick, P Martin [principal investigator]). K Varty Birmingham Heartlands Hospital, Birmingham (D Adam, J Bell, A W Bradbury, P Crowe, M Gannon, M J Henderson, D Sandler, R A Shinton [principal investigator], J M Scriven, T Wilmink). Lancashire Teaching Hospitals NHS Trust, Preston (S D'Souza, A Egun, R Guta, S Punekar, D M Seriki [principal investigator], G Thomson). Liverpool Royal Infirmary and the Walton Centre, Liverpool (J A Brennan, T P Enevoldson, G Gilling-Smith [principal investigator], D A Gould, P L Harris,R G McWilliams, H-C Nahser, R White). Manchester Royal Infirmary, Manchester (K G Prakash, F Serracino-Inglott, G Subramanian [principal investigator], J V Symth, M G Walker). Newcastle Acute Hospitals NHS Foundation Trust, Newcastle upon Tyne (M Clarke, M Davis, S A Dixit, P Dorman [principal investigator], A Dyker, G Ford, A Golkar, R Jackson, V Jayakrishnan, D Lambert, T Lees, S Louw, S Macdonald, A D Mendelow, H Rodgers, J Rose, G Stansby, M Wyatt). North Bristol NHS Trust, Frenchay Hospital, Bristol (T Baker, N Baldwin [principal investigator], L Jones, D Mitchell, E Munro, M Thornton). Royal Free Hospital, London (D Baker, N Davis, G Hamilton [principal investigator], DJH McCabe, A Platts, J Tibballs). Sheffield Teaching Hospitals NHS Foundation Trust, Sheffield (J Beard, T Cleveland, D Dodd, P Gaines, R Lonsdale, R Nair, A Nassef, S Nawaz, G Venables [principal investigator]). St George's University of London and St George's NHS Healthcare Trust, London (A Belli, A Clifton, G Cloud, A Halliday, H Markus [principal investigator], R McFarland, R Morgan, A Pereira, A Thompson). St Mary's Hospital, Imperial College Healthcare NHS Trust, London (J Chataway

[principal investigator], N Cheshire, R Gibbs, M Hammady, M Jenkins, I Malik, J Wolfe). University College London Hospitals NHS FoundationTrust, London (M Adiseshiah, C Bishop, S Brew, J Brookes, M M Brown [principal investigator], R Jäger, N Kitchen). University Hospital of South Manchester, Wythenshawe, Manchester (R Ashleigh, S Butterfield, G E Gamble, C McCollum [principal investigator], A Nasim, P O'Neill, J Wong). Western Infirmary, Glasgow (R D Edwards, K R Lees, A J MacKay, J Moss [principal investigator], P Rogers).

Methods for time-updated Cox proportional hazards models

To model the effect of restenosis on subsequent stroke or vascular death, we used Cox proportional hazards models with a time updated covariate, a well-established statistical method (see for example “Andersen, Encylopedia of Biostatistics, 2005, Chapter: Time-Dependent covariates”). This is achieved by splitting records for an individual into relevant segments of follow up and then running a proportional hazards model on the modified data.

For example, suppose a patient has restenosis after 69 days and subsequently has a stroke after 300 days. Their original record is:

Subject ID	Stroke	Start of Follow up	End of follow up	Time to restenosis
1	1	0	300	69

To fit a model with a time-varying covariate the data are modified to:

Subject ID	Stroke	Start of Follow up	End of follow up	Time to restenosis	After restenosis
1	0	0	69	69	0
1	1	69	300	69	1

The covariate in the “after restenosis” column can now be used to compare the rate of stroke before and after restenosis. From a technical standpoint, the new hazard function becomes:

$$h_{0i}(t) = \exp(\propto (t) + \beta_1 x_{i1} + \beta_2 x_{i2}(t))$$

where $\propto (t)$ is the baseline hazard function, x_{i1}represents a “normal” covariate (i.e. one that does not change over time), and $x_{i2}(t)$ represents a covariate that is allowed to vary over time. β_2 then represents the log hazard ratio per unit change in the time-dependent covariate.

Further explanation can be found in “Andersen, Encylopedia of Biostatistics,2005, Chapter: Time-Dependent covariates”. The method is readily implemented with a range of statistical software (e.g. “stsplit” in Stata, “survival” package in R).

Supplementary Table 1: Duplex ultrasound velocity criteria used for grading the degree (%) of carotid stenosis.

The degree of stenosis is expressed in values considered equivalent to NASCET angiography measures. PSV indicates peak systolic velocity; EDV, end diastolic velocity; ICA, internal carotid artery; CCA, common carotid artery. Velocity measurements are in meters/second. In cases where the flow velocity criteria spanned more than one category of stenosis, the higher category of stenosis was chosen if the PSV ICA and either the EDV ICA or the PSV ICA / PSV CCA ratio were within the higher band.

**Band of stenosis (%)**	**PSV ICA (m/s)**	**EDV ICA (m/s)**	**PSV ICA / PSV CCA**
0 – 29	<1·1	<0·4	<3·2
30 – 49	1·1 – 1·3	<0·4	<3·2
50 – 59	>1·3 – 2·1	<0·4	<3·2
60 – 69	>1·3 – 2·1	0·4 – 1·1	3·2 – 4·0
70 – 79	>2·1	>1·1 – 1·4	>4·0
80 – 95	>2·1	>1·4	>4·0
96 – 99	String Flow	String Flow	String Flow
100	Occluded	Occluded	Occluded

Supplementary Table 2: Multivariate predictors of restenosis chosen using forward stepwise variable selection

	HR	95% CI		P-value
**Risk factors for ≥50% restenosis**				
Stenting (vs. endarterectomy)	1.38	1.14	1.69	0·001
Degree of contralateral stenosis				<0·0001
50-69%	1·50	1·15	1·95	
>70%	2·25	1·73	2·92	
Occlusion	3·06	2·09	4·49	
Female sex	1·54	1·25	1·91	<0·0001
Cholesterol (per mmol/l)	0·87	0·80	0·94	0·001
Systolic blood pressure (per mmHg)	1·01	1·01	1·02	<0·0001
Diastolic blood pressure (per mmHg)	0·99	0·98	1·00	0·006
Smoking status				<0·0001
Current smoker	2·22	1·64	2·98	
Ex-smoker	1·68	1·30	2·16	
Non-insulin dependent diabetes	1·35	1·05	1·74	0·018
Age (per year)	1·01	1·00	1·03	0·027
History of angina	1·53	1·12	2·08	0·007
**Risk factors for ≥70% restenosis**				
Stenting (vs. endarterectomy)	1.17	0.83	1.67	0·36
Degree of contralateral stenosis				<0·0001
50-69%	1·14	0·68	1·91	
>70%	2·17	1·40	3·36	
Occlusion	2·23	1·21	4·12	
Non-insulin dependent diabetes	1·60	1·06	2·40	0·025
